# Evaluation of a culture change program to reduce unprofessional behaviours by hospital co-workers in Australian hospitals

**DOI:** 10.1186/s12913-024-11171-0

**Published:** 2024-06-12

**Authors:** Johanna I. Westbrook, Rachel Urwin, Ryan McMullan, Tim Badgery-Parker, Antoinette Pavithra, Kate Churruca, Neil Cunningham, Erwin Loh, Peter Hibbert, Guy Maddern, Jeffrey Braithwaite, Ling Li

**Affiliations:** 1 Australian Institute of Health Innovation, Macquarie University, Sydney, NSW Australia; 2 Vincent’s Hospital Melbourne, Melbourne, VIC Australia; 3 Discipline of Surgery, University of Adelaide, Adelaide, South Australia Australia

**Keywords:** Incivility, Bullying, Disruptive behaviours, Professionalism, Speaking-up, Workplace mistreatment

## Abstract

**Background:**

Unprofessional behaviours between healthcare workers are highly prevalent. Evaluations of large-scale culture change programs are rare resulting in limited evidence of intervention effectiveness. We conducted a multi-method evaluation of a professional accountability and culture change program “Ethos” implemented across eight Australian hospitals. The Ethos program incorporates training for staff in speaking-up; an online system for reporting co-worker behaviours; and a tiered accountability pathway, including peer-messengers who deliver feedback to staff for ‘reflection’ or ‘recognition’. Here we report the final evaluation component which aimed to measure changes in the prevalence of unprofessional behaviours before and after Ethos.

**Methods:**

A survey of staff (clinical and non-clinical) experiences of 26 unprofessional behaviours across five hospitals at baseline before (2018) and 2.5–3 years after (2021/2022) Ethos implementation. Five of the 26 behaviours were classified as ‘extreme’ (e.g., assault) and 21 as incivility/bullying (e.g., being spoken to rudely). Our analysis assessed changes in four dimensions: work-related bullying; person-related bullying; physical bullying and sexual harassment. Change in experience of incivility/bullying was compared using multivariable ordinal logistic regression. Change in extreme behaviours was assessed using multivariable binary logistic regression. All models were adjusted for respondent characteristics.

**Results:**

In total, 3975 surveys were completed. Staff reporting frequent incivility/bullying significantly declined from 41.7% (*n* = 1064; 95% CI 39.7,43.9) at baseline to 35.5% (*n* = 505; 95% CI 32.8,38.3; χ^2^(1) = 14.3; *P* < 0.001) post-Ethos. The odds of experiencing incivility/bullying declined by 24% (adjusted odds ratio [aOR] 0.76; 95% CI 0.66,0.87; *P* < 0.001) and odds of experiencing extreme behaviours by 32% (aOR 0.68; 95% CI 0.54,0.85; *P* < 0.001) following Ethos. All four dimensions showed a reduction of 32–41% in prevalence post-Ethos.

Non-clinical staff reported the greatest decrease in their experience of unprofessional behaviour (aOR 0.41; 95% CI 0.29, 0.61). Staff attitudes and reported skills to speak-up were significantly more positive at follow-up. Awareness of the program was high (82.1%; 95% CI 80.0, 84.0%); 33% of respondents had sent or received an Ethos message.

**Conclusion:**

The Ethos program was associated with significant reductions in the prevalence of reported unprofessional behaviours and improved capacity of hospital staff to speak-up. These results add to evidence that staff will actively engage with a system that supports informal feedback to co-workers about their behaviours and is facilitated by trained peer messengers.

**Supplementary Information:**

The online version contains supplementary material available at 10.1186/s12913-024-11171-0.

## Introduction

Unprofessional behaviour in healthcare is widespread [1–3] and significantly undermines the effective functioning of teams, staff wellbeing, patient experience and safety, and negatively impacts organisational productivity and costs [3–8]. Unprofessional behaviour encompasses a spectrum, from overtly hostile, bullying and inappropriate behaviours such as physical and verbal abuse, to more subtle behaviours such as passive aggression, rudeness and incivility [3, 9]. Consistently high rates of incivility and bullying between healthcare co-workers have been demonstrated in surveys from multiple countries and settings [2, 10–12].

Many organisations are seeking effective interventions to reduce unprofessional behaviours and improve organisational cultures. However, as exemplified in a recent systematic review [13], a very limited number of large-scale organisational culture change interventions have been described in detail or robustly evaluated [6, 13, 14].

The Promoting Professional Accountability Program [3, 15] developed by Vanderbilt University Medical Center, has been the most widely adopted model designed to target and reduce unprofessional behaviours. The program is based upon the use of graded interventions for primarily medical staff [3, 16]. Core elements include the Patient Advocacy Reporting System (PARS) and the Co-worker Observation Reporting System (CORS), which allow patients and clinical staff to report concerns and complaints about unprofessional behaviours. These concerns are shared as informal feedback with the subjects of the reports by trained peer-messengers, and data on patterns of complaints are used to demonstrate that an individual’s behaviour is outside the boundaries of their peers (e.g., number of patient or co-worker complaints), with the aim of encouraging reflection and behaviour change [17]. If poor behaviours persist, then leader-initiated plans to support the individual are developed. The use of disciplinary processes is seen as the last resort when plans fail [15].

Early research on this approach demonstrated that most medical staff were receptive to conversations with peers [16]. A study across three large US hospitals which examined patterns of co-worker complaints over time found that 71% of individuals who received peer messenger feedback had no further reports in the following year [17]. Data generated from the CORS database have also more recently been used to investigate the relationships between frequent co-worker and patient complaints, and poor patient safety outcomes (e.g., mortality and post-operative complications) [4, 18]. In a study of 13,653 patients treated by 202 surgeons, patients whose surgeons had higher numbers of co-worker reports were at significantly greater risk of post-operative complications than patients with surgeons with fewer co-worker complaints [4]. Such results further reinforce the imperative to implement effective interventions to reduce these behaviours. Despite increased adoption of professional accountability programs [17, 19–22], evidence of their effectiveness to drive organisational-wide culture change and significantly reduce the overall prevalence of unprofessional behaviours is absent.

In 2017, St Vincent’s Health Australia developed the Ethos professional accountability and culture change program [21, 23] which adapted and extended the Vanderbilt professional accountability model. The aim of the Ethos program is to support improvements in organisational culture with a specific focus on reducing rates of unprofessional behaviour between staff. The Ethos program comprises multiple components including staff capability training (e.g., a graded assertiveness to encourage staff to speak-up in the moment when they experience or witness behaviours that undermine a culture of safety). An online submission system allows all hospital staff to anonymously report unprofessional behaviours (delivered to individuals as ‘feedback for reflection’), as well as positive staff behaviours that enhance safety and care delivery (‘feedback for recognition’). Submissions are triaged by a small team of trained staff who review submissions and allocate trained peer-messengers to deliver the reflection messages. Peer messengers receive training and ongoing support in their role in delivering messages to colleagues [24]. Ethos messages are designed to provide information about the perceived impact of the recipient’s behaviour on others and an opportunity to reflect. Messages that include accounts of serious unprofessional behaviour, such as physical or sexual assault, are escalated to human resources as part of existing formal disciplinary review processes. Analysis of submissions allows for the identification of staff who demonstrate a pattern of unprofessional behaviour which prompt additional actions towards perpetrators, including staff welfare checks, development of a management plan and disciplinary procedures. Submissions for recognition are forwarded to the subject’s supervisor to deliver. Further details of the program [25] and implementation have been published [21, 26].

We designed a multi-component evaluation program to assess the use, challenges and effects of the Ethos Program [24, 26, 27]. This evaluation included detailed analysis of the reports submitted by staff to the Ethos messaging system [28], which revealed widespread use of the system by all professional groups both for recognising positive and reporting unprofessional behaviour for reflection. Analysis of 2504 submissions showed 47.7% were for recognition and 52.3% for reflection. All professional groups were represented in senders and receivers of messages, with nurses being the greatest users (20.1 submissions/100 nurses across eight hospitals), and non-clinical staff the lowest (5.1/100 staff). All staff groups were identified as the subject of Ethos messages, with rates ranging from 19.5 messages/100 medical staff to 10.5/100 non-clinical staff. A survey of peer-messengers trained to deliver messages of reflection to co-workers identified both practical issues, e.g., finding a convenient time and place to meet, as well as some of the challenges of having difficult conversations with colleagues [24].

A widespread training program for staff in speaking-up was a core element of the program. The Ethos messaging system was only designed to be used when staff felt unable to speak-up in the moment or to talk with their supervisors. Interviews with middle managers identified the critical role that they play in enacting and operationalising the Ethos program. How middle managers respond when staff speak-up about unprofessional behaviours by co-workers is an essential element in supporting improved psychological safety [24]. Staff do not always want actions to be taken but want to be heard when they speak-up. However, this can transfer new responsibilities to these middle managers [27].

To ensure that we could assess the impact of the Ethos program on the overall prevalence of unprofessional behaviour, we conducted a survey of staff experiences at baseline to quantify the extent and nature of the problem [29]. This survey asked about 26 behaviours which ranged from ‘having opinions ignored’ to ‘physical assault’. Open-ended questions also asked staff to provide accounts of their experiences at their hospital and 32% (*n* = 1636) of respondents provided detailed narratives. Analyses revealed the negative impacts these behaviours had on individuals, as well as on patient care and safety [30].

As the final component of the evaluation program, our aim was to repeat the prevalence survey 2.5 to 3 years after the Ethos program had been in place to determine whether the prevalence and types of unprofessional behaviour had changed and to assess staff awareness, views, and use of the program. Due to the COVID-19 pandemic, only five hospitals could be included in the follow up survey.

## Methods

### Study design and participants

We conducted a pre–post cross-sectional study across five metropolitan hospitals in two Australian states by inviting all staff to complete an anonymous online survey. During a two-week period, researchers attended wards, departments and common areas (including weekends/nightshifts) to encourage participation and offer incentives (e.g., $50 daily gift-card prize-draw).

Baseline prevalence of unprofessional behaviours, prior to Ethos program implementation, was obtained by administration of the Longitudinal Investigation Of Negative behaviour (LION) survey between December 2017 and November 2018 at seven hospitals [29]. We repeated the survey between October 2021–February 2022, which was on average 2.5 to 3 years after Ethos implementation. Due to the COVID-19 pandemic, only five hospitals were included in the follow-up study. The baseline surveys for these five hospitals were conducted between July–November 2018. A minimum sample of 368 surveys was required (staff population *n* = 8373, with 95% confidence, 5% error margin for 50% estimated proportion).

### Materials

The LION survey [29] examines 26 unprofessional behaviours from incivility (e.g., being spoken to rudely) to assault, experienced in the previous 12-months. The survey incorporates questions from the Negative Acts Questionnaire Revised (NAQ-R) [31] and the Royal Australasian College of Surgeons Discrimination, Bullying and Sexual Harassment Survey [29]. Participants rate the frequency with which they experienced each behaviour using a 7-point scale from never to multiple times a day (Supplementary File-1).

Questions about ‘speaking-up’ and demographics including age, gender, professional group (i.e., medical; nursing; allied health and clinical services); non-clinical services (e.g., scientist, cleaners); management and administrative (e.g., finance, ward clerks); and length of employment at the hospital and in the health sector are included. The survey was piloted and tested for face validity. The follow-up survey included questions about the seniority of perpetrators of unprofessional behaviours, knowledge and views of the Ethos program, and the impact of COVID-19 on unprofessional behaviours (Supplementary File-1).

Human Research Ethics approval was granted by St Vincent’s Hospital Melbourne Human Research Ethics Committee for a multi-site study (HREC/17/SVHM/237). All participants in the study provided informed consent, and provided their agreement to participate by clicking on “I agree” to participate in the survey before proceeding.

### Statistical analysis

Response rates were calculated using staffing numbers from each hospital. In the follow-up period we were unable to adjust the denominator to account for all staff absent due to the COVID-19 pandemic, and thus the response rate is likely to be an underestimate. Surveys with at least 60% of questions answered (these made up 90% of all surveys at baseline and follow-up) were analysed. Of included surveys, average completion was > 95% of questions (Supplementary File-1). Characteristics of baseline and follow-up samples were compared using χ^2^ tests. We grouped five of the 26 unprofessional behaviours as ‘extreme’ (physical assault, threats of violence, inappropriate/unwanted touching, demands for sexual favours, and sexual assault) and 21 as incivility/bullying behaviours. The five ‘extreme’ behaviours were classified as ‘Ever’ if experienced in the past 12-months or ‘Never.’ The 21 ‘incivility/bullying’ behaviours were grouped into: ‘Never’ in the past year, ‘Occasional’ at least one behaviour 1–2 times/year to monthly, and ‘Frequent’ if at least one behaviour was experienced weekly to multiple times daily [29]. Missing data were reported in descriptive statistics where applicable.

Experience of incivility/bullying was compared using multivariable ordinal logistic regression. This method models the odds of being in a higher category compared with the odds of being in a lower category, i.e., ‘Frequent/Occasional’ vs ‘Never’, or ‘Frequent’ vs ‘Occasional/Never’, with the proportional odds assumption ensuring that the odds ratios (ORs) are the same across categories. In these models, an OR of less than 1.0 indicates that staff in the follow-up period had reduced odds of reporting high frequencies of incivility/bullying behaviours compared to staff in the baseline. Experience of extreme unprofessional behaviour at baseline and follow-up was compared using multivariable binary logistic regression, i.e., modelling ‘Ever’ vs ‘Never’. All models were adjusted for age, gender, role, hospital, and length of employment in hospital and in the health sector. Separate multivariable ordinal logistic regression models were fit for each hospital to estimate changes in unprofessional behaviour. A role-by-survey interaction term was added to the overall model with all hospitals and used to estimate changes for each role. Complete case analysis was applied in each model.

The 26 unprofessional behaviours were mapped to four dimensions: work-related bullying; person-related bullying; physical bullying and sexual harassment [31] (Supplementary file-1). Adjusted odds ratios for each of the four dimensions were calculated, using multivariable logistic regression models based on ‘ever’ or ‘never’ experiencing any unprofessional behaviours assigned to each dimension.

For Likert-type items, differences between surveys were tested using χ^2^ tests and multinomial simultaneous confidence intervals calculated [32]. Analyses were conducted using SAS version 9.4 and R version 4.2.

## Results

### Characteristics of respondents

The response rate was 30.7% (*n* = 2552/8320) at baseline and 17.0% (*n* = 1423/8373) at follow-up (Supplementary-1). Respondents at baseline and follow-up were similar in age distribution, length of time employed at their hospital and in the health sector (Table 1). At follow-up, significantly fewer medical staff completed the survey and Hospital A staff made up a smaller proportion. The reduced medical sample accounted for a gender difference between survey samples. At baseline, 43% of medical respondents were female, compared with 59% at follow-up. When medical respondents were excluded, there was no gender difference (χ^2^(1) = 0.83; *P* = 0.36) between groups.

**Table 1:** Summary of the characteristics of respondents in the baseline and follow-up surveys

Variable	Baseline (*N* = 2552)		Follow-up (*N* = 1423)		*P* value^*^
	n	%	n	%	
**Age group (years)**					
18–24	122	4.8	69	4.8	0.35
25–34	715	28.0	376	26.4	
35–44	583	22.8	346	24.3	
45–54	547	21.4	325	22.8	
55 +	528	20.7	265	18.6	
Prefer not to answer	55	2.2	40	2.8	
Missing	2	0.1	2	0.1	
**Gender**					
Male	613	24.0	279	19.6	0.002
Female	1884	73.8	1108	77.9	
Other	5	0.2	3	0.2	
Prefer not to answer	50	2.0	31	2.2	
Missing	0	0	2	0.1	
**Role**					
Nursing	1035	40.6	637	44.8	< 0.001
Medical	302	11.8	75	5.3	
Allied Health & Clinical Services	371	14.5	214	15.0	
Non-clinical Services	337	13.2	211	14.8	
Management & Administrative	423	16.6	284	20.0	
Missing	84	3.3	2	0.1	
**Hospital**					
A	1309	51.3	536	37.7	< 0.001
B	300	11.8	168	11.8	
C	371	14.5	275	19.3	
D	430	16.8	316	22.2	
E	142	5.6	128	9.0	
**Length Employed at Hospital (years)**					
< 1	318	12.5	219	15.4	0.08
1–2	387	15.2	201	14.1	
3–5	594	23.3	312	21.9	
6–10	504	19.7	276	19.4	
11–20	510	20.0	304	21.4	
20 +	227	8.9	109	7.7	
Missing	12	0.5	2	0.1	
**Length Employed in Sector (years)**					
< 1	106	4.2	72	5.1	0.08
1–2	180	7.1	104	7.3	
3–5	390	15.3	198	13.9	
6–10	513	20.1	297	20.9	
11–20	566	22.2	361	25.4	
20 +	767	30.1	385	27.1	
Missing	30	1.2	6	0.4	

^*^χ^2^ test, excluding Missing, Prefer not to answer, and Other categories

### Changes in prevalence of four dimensions of unprofessional behaviour

We examined changes in four dimensions of unprofessional behaviour: work related bullying; person-related bullying; physical bullying and sexual harassment (Table 2). The adjusted odds ratios (aORs) showed reported behaviours in each dimension significantly declined by a minimum of 30% in the post period.

**Table 2:** Adjusted odds ratios for experiencing unprofessional behaviour (Ever vs Never) of post-ethos compared to pre-ethos

Dimensions of Unprofessional Behaviours	AOR (95% CI))	*P*-value
Work related bullying	0.68 (0.56, 0.81)	** < 0.001**
Person-related bullying	0.66 (0.54, 0.80)	** < 0.001**
Physical bullying	0.66 (0.57, 0.76)	** < 0.001**
Sexual harassment	0.59 (0.50, 0.68)	** < 0.001**

Adjusted for staff age, gender, role, hospital, and length of employment in the hospital

### Changes in prevalence of incivility/bullying behaviours

At baseline across all hospitals, 6.4% (95% CI 4.3,8.5) of respondents reported they had ‘Never’ experienced any of the 21 incivility/bullying behaviours in the previous 12-months. This significantly increased to 11.8% (95% CI 9.1,14.6; χ^2^(1) = 33.6; *P* < 0.001) at follow-up.

Staff reporting frequent incivility/bullying (i.e., ≥ one of these 21 behaviours weekly or more frequently) significantly declined from 41.7% (95% CI 39.7,43.9) to 35.5% (95% CI 32.8,38.3; χ^2^(1) = 14.3; *P* < 0.001). After adjusting for age, gender, role, hospital, length of employment in their hospital and sector, staff in the follow-up period experienced incivility/bullying significantly less frequently than at baseline (aOR 0.76; 95% CI 0.66,0.87; *P* < 0.001). The odds of experiencing higher frequency of incivility/bullying behaviours in the last 12-months were 24% lower in the follow-up period compared to baseline.

At each of the five hospitals the odds of being more likely to experience incivility/bullying at follow-up compared to baseline decreased (Table 3). This decrease was statistically significant at Hospital A (aOR 0.76; *P* = 0.01) and Hospital C (aOR 0.55; *P* < 0.001). Excluding medical respondents made little difference to any of these hospital estimates (Supplementary-2).

**Table 3:** Odds ratio for experiencing incivility/bullying at each hospital, follow-up vs baseline

Hospital	Number of responses (baseline and follow-up)	Unadjusted analysis			Adjusted analysis^a^		
		OR	95% CI	*P*	OR	95%CI	*P*
Hospital A – Major teaching hospital with ED	1672	0.73	0.59, 0.90	0.003	0.76	0.62, 0.94	0.01
Hospital B – Medium private hospital	417	0.73	0.50, 1.08	0.12	0.74	0.49, 1.12	0.15
Hospital C – Large private hospital	600	0.73	0.53, 1.00	0.05	0.55	0.39, 0.78	< 0.001
Hospital D – Medium Private hospital	670	0.85	0.63, 1.14	0.29	0.79	0.58, 1.08	0.14
Hospital E – Small Private hospital	242	0.81	0.50, 1.30	0.38	0.79	0.48, 1.30	0.34

*ORs* Odds ratios are from ordinal logistic regression models comparing odds of being more likely to experience unprofessional behaviour (i.e., ‘Frequent/Occasional’ vs ‘Never’ or ‘Frequent’ vs ‘Occasional/Never’) at follow-up compared with baseline

^a^Adjusted for age, gender, role, and length of employment in the hospital and in the sector

Non-clinical services staff at follow-up reported the greatest reduction (59%) in odds of experiencing higher frequency of incivility/bullying (aOR 0.41; *P* < 0.001) compared to baseline, followed by management and administrative staff who experienced a 34% reduction (aOR 0.66; *P* = 0.009; Table 4). Odds ratios for staff in other roles were not statistically significant, but the point estimates all showed a decrease.

**Table 4:** Odds ratio for experiencing incivility/bullying by role, follow-up vs baseline

Role	Number of responses (baseline and follow-up)	Unadjusted analysis			Adjusted analysis^a^		
		OR	95% CI	*P*	OR	95% CI	*P*
Nursing	1548	0.83	0.69, 1.02	0.08	0.89	0.73, 1.09	0.26
Medical	353	1.06	0.64, 1.76	0.83	0.98	0.58, 1.64	0.94
Allied Health & Clinical Services	552	0.76	0.54, 1.07	0.12	0.86	0.61, 1.21	0.38
Non-clinical Services	494	0.36	0.25, 0.53	< 0.001	0.41	0.29, 0.61	< 0.001
Management & Administrative	654	0.57	0.42, 0.78	< 0.001	0.66	0.48, 0.90	0.009

*ORs* Odds ratios are from an ordinal logistic regression model comparing odds of being more likely to experience unprofessional behaviour (i.e., ‘Frequent/Occasional’ vs ‘Never’ or ‘Frequent’ vs ‘Occasional/Never’) at follow-up compared with baseline

^a^Adjusted for age, gender, hospital, and length of employment in the hospital and in the sector

For 17 of the 21 incivility/bullying behaviours there was a significant reduction in prevalence at follow-up (Supplementary-3). The remaining four showed no change.

### Changes in the prevalence of extreme unprofessional behaviours

Respondents reporting ‘Ever’ experiencing extreme behaviours in the previous 12-months decreased significantly from 14.6% (*n* = 371; 95% CI 13.3,16.1) at baseline to 10.0% (*n* = 141 95% CI 8.5,11.7; *P* < 0.001) at follow-up. The decrease was similar by gender and age. After adjusting for respondent characteristics, the odds of experiencing extreme behaviours remained significantly lower (aOR 0.68; 95% CI 0.54,0.85; *P* < 0.001) at follow-up, demonstrating a 32% overall reduction in the odds of experiencing extreme behaviours compared to baseline.

The overall reduction in extreme behaviours was driven particularly by results from Hospitals A and D. These results were similar when medical respondents were excluded from both baseline and follow-up samples (Supplementary-4).

### Seniority of perpetrators and changes in unprofessional behaviours during COVID-19

For 23 of the 26 behaviours the perpetrator was more senior than the victim in the majority of instances. For the remaining three (physical assault; being shown suggestive photos/videos or texts; unwelcome sexual flirtations, requests for dates) the perpetrator was either the same level or more senior in the majority of instances (Supplementary-5).

Of staff who responded to the COVID-19 question (*n* = 1318), 64.4% (95% CI 61.8,67.1) reported no change in unprofessional behaviours during COVID-19, 23.8% (95% CI 21.2,26.5) an increase and 11.8% (95% CI 9.2,14.5) a decrease.

### Changes in staff attitudes about speaking-up and reporting unprofessional behaviours

**Fig. 1:**
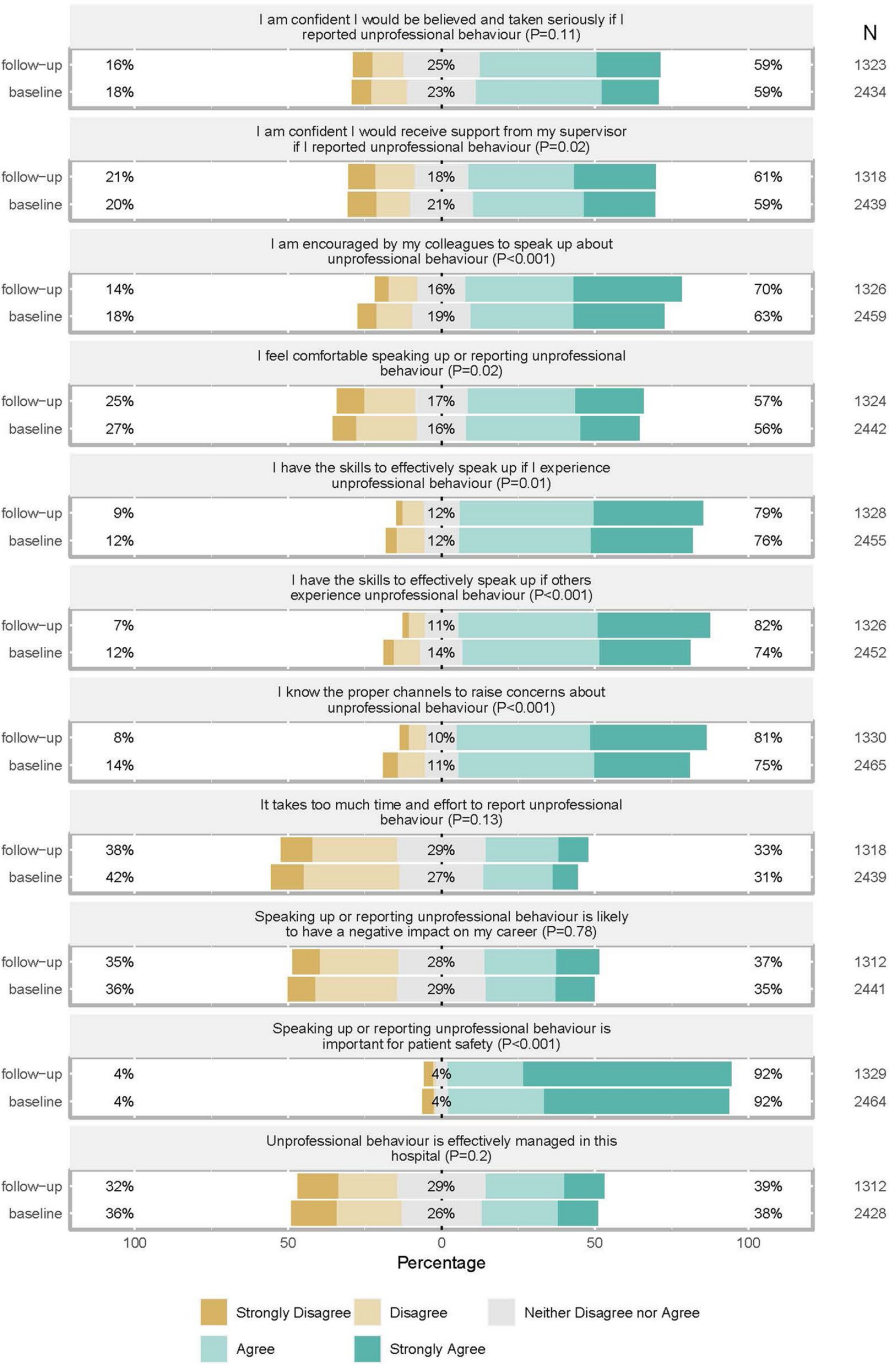
Respondents’ views about speaking-up and reporting unprofessional behaviour, baseline and follow-up

Staff were asked to indicate their level of agreement to 11 items related to speaking-up and reporting. For seven items agreement significantly improved, indicating more positive views about speaking-up and reporting at follow-up (Fig. 1, Supplementary File-6). Two items asked respondents about their skills to effectively speak-up if they, or others, experienced unprofessional behaviours. Agreement to these skill questions increased significantly at follow-up (Fig. 1).

### Staff awareness, use and perceptions of impact of the Ethos program

At follow-up, 82.1% (95% CI 80.0,84.0) of respondents were aware of the Ethos program. Of these 1168, 79.9% (95% CI 77.5,82.1) had seen promotional material and 63.9% (95% CI 61.1,66.6) had completed Ethos training. In total 23.9% (95% CI 21.5,26.4; *n* = 279) had sent an Ethos message and 9.4% (95% CI 7.9,11.2%) had received a message. Most messages received (71%) were for recognition, with 29% for reflection. Overall, 16% (*n* = 186) of respondents aware of the program reported that Ethos had changed the way they interact with other staff.

Of those who reported knowledge of the Ethos program (*n* = 1168), 29.7% (95% CI 26.6,32.9; *n* = 347) indicated that Ethos was slightly/completely effective; 26.4% (95% CI 23.4,29.6; *n* = 309) not very/not at all effective, 37.2% (95% CI 34.1,40.3; *n* = 434) were not sure, and 78 did not respond.

Of nine areas of possible impact, respondents provided the greatest support for Ethos having an impact on the ability of others to speak-up, to speak-up themselves and to speak-up for others. Respondents’ views on perceived impact for the remaining six areas, from teamwork to the frequency of errors, was mixed. On average around a third reported some positive impact, a third were unsure, and the remainder reported no impact (Supplementary-7).

We examined respondents’ ratings of the effectiveness of the Ethos program based upon their involvement in the program. We found that those who had sent a message rated Ethos as more effective than those who had not (42.8% [95% CI 36.2,48.9] versus 27.7% [95% CI 23.4,30.9]). Respondents who had received an Ethos message were more likely to report Ethos as being effective relative to staff who had not received a message (49.6% [95% CI 40.0,58.7] versus 29.6% [95% CI 25.6,32.4]). Staff who had received a recognition message were more likely to think Ethos was effective compared to those who had received a message for reflection (56.3% [95% CI 46.3,68.1] versus 37.5% [95% CI 21.2,56.2]). However, respondents who had received a message for reflection were more likely to think Ethos was effective compared to those who had received no Ethos message (37.5% [95% CI 21.2,56.2] versus 29.6% [95% CI 25.6,32.4]).

## Discussion

We found that 2.5–3 years after the implementation of the Ethos program across five hospitals, the prevalence of unprofessional behaviours reported by staff significantly declined. The odds of experiencing incivility/bullying behaviours decreased by 24%, with significant reductions in the prevalence of 17 of 21 incivility/bullying behaviours. There was a 32% reduction in odds of extreme unprofessional behaviours in the previous 12-months, with significant declines in each of the four dimensions of unprofessional behaviours. Staff attitudes to speaking-up and expectations when they reported unprofessional behaviours were significantly more positive at follow-up. Respondents were also most likely to identify speaking-up skills for themselves and others as an impact of the Ethos program.

The effects were not uniform across each of the five hospitals when examined individually. While we adjusted for hospital differences in our analyses of the overall changes in incivility/bullying behaviours post-Ethos, we did not power our study to be able to examine individual hospital differences. The two largest hospitals (A and C) showed a significant decline in incivility/bullying compared to the other smaller hospitals. However, it was interesting to note that all hospitals showed a decline in their odds ratios. The smallest hospitals (D and E) had the Ethos program in place for the shortest period at the time of follow-up (i.e., one year shorter than for Hospital A and B; 6-months shorter than for Hospital C). Future research examining differences over time and between hospitals with different contexts, as well as the implementation processes would be of value in understanding the extent to which the program generates consistent effects.

No previous evaluation of professional accountability programs has measured prevalence of unprofessional behaviours before and after implementation [13]. Evaluations of professional accountability programs in the US have focused on assessing the impact on individuals, primarily doctors, who have received messages about their unprofessional behaviours [16, 17]. These studies have shown that most staff who receive peer-messages are not named in subsequent reports [17], suggesting interventions are effective in influencing individuals’ future behaviours. Our finding of reduced rates of unprofessional behaviours experienced across a large hospital population is consistent with this effect.

However, given that only a relatively small proportion of staff receive a message for ‘reflection’ [21], our results importantly suggest that the reduction in unprofessional behaviours may also be attributable to behaviour change in those who did not receive messages for reflection. Multiple mechanisms may have played a role. Our results suggest that formal parts of the Ethos program, such as training, were effective in building capacity in speaking-up, with staff reporting higher levels of skills at follow-up compared to baseline. Informal communication between staff about their direct and indirect experiences of sending and receiving Ethos messages, along with the presence of multiple staff trained as peer-messengers, who act as champions for the program, are also likely to have contributed to signalling heightened organisational expectations of acceptable behaviours. Our other evaluation methods including surveys with peer messengers [24] and interviews with middle managers [27] provide some insights into these mechanisms for cultural change.

As reported by other studies [33], we found the perpetrators of unprofessional behaviour were most often senior to the victim. This finding reinforces the importance of ensuring that senior staff are engaged in any culture change program in order to bring about significant reductions in unprofessional behaviours. Ensuring senior staff model professional behaviours including being seen by more junior staff to speak-up in the moment wherever possible, has been shown to be particularly important in shaping young doctors’ behaviours [34]. Training senior staff as messengers and providing them with the skills to address unprofessional behaviour, as well as rewarding staff for positive behaviour are all strategies which are incorporated into the Ethos program, but there will be local variation in the extent to which these strategies are effective and sustained. As a recent realist review [9] has identified, the problem of unprofessional behaviour needs to be tackled in a holistic way recognising a multitude of factors in different contexts.

Unlike previously reported professional accountability programs, the Ethos program encourages messages for ‘recognition’ and these make up approximately 50% of all messages submitted [21]. These positive messages may also have contributed to reduced rates of unprofessional behaviours. Other studies have demonstrated that positive staff recognition programs are associated with improved work performance and well-being [35–38]. Limited attention has been paid to the role that positive feedback from colleagues at scale may play in driving better organisational cultures, which is a key feature of the Ethos program, one well utilised by staff, and this is worthy of further investigation.

This is the first study to report the impact of professional accountability programs on non-clinical staff, and it was this group that experienced the greatest reductions in their experience of unprofessional behaviours. These findings suggest an important deficit in other programs which attempt to address unprofessional behaviour in professional silos, with a particular focus on targeting unprofessional behaviour among doctors and nurses. Our evaluation of the online messages showed that clinical staff had the highest rates of using the online messaging system. However, all professional groups submitted Ethos messages about unprofessional behaviours at roughly equal inter- and intra-professional rates, which is not surprising given the multidisciplinary nature of healthcare [28]. The reason we found the greatest decline in unprofessional behaviours experienced by non-clinical and managerial staff may be related to the fact that these groups had not previously been incorporated into targeted programs to reduce unprofessional behaviours. The mechanism may well have been a change in behaviours across professional groups towards non-clinical staff triggered by the Ethos program. This is an area that would be interesting to explore in future studies. As noted, we had a lower response rate from doctors in the post-Ethos survey and this may also explain a reduced impact in this group.

Unprofessional behaviours by medical and nursing staff have been shown to be associated with poorer clinical outcomes [4, 6, 18, 39]. We do not have equivalent studies of non-clinical hospital staff and safety outcomes. Given the level of collaboration and coordination required between clinical and non-clinical staff to provide safe patient care, it seems reasonable to hypothesise that unprofessional behaviour both within and between professional groups are likely contributors to reduced safety. Our results reinforce the potential value of implementing programs which target unprofessional behaviours in all staff groups in hospitals to achieve improvements in organisational cultures.

### Strengths and limitations

As we were unable to conduct a controlled before-and-after study, consideration needs to be given to factors other than the Ethos program which may have influenced the findings. The most noteworthy was the COVID-19 pandemic. Each of the study hospitals was affected in multiple, but different, ways (e.g., managing COVID-19 patients, staff shortages, shift to new modes of care, job insecurity due to reduced elective surgery). The rates of transmission of COVID-19 were also different between the two states [40]. Thus, staff were under considerable pressure, particularly at the largest hospital, which had a high number of COVID-19 patients and an emergency department. Resource shortages and work demands have been implicated as triggers for unprofessional behaviours [19, 41]. A survey [42] of 526 US nurses conducted between June–September 2020 found 37.4% reported greater co-worker incivility during the COVID-19 pandemic. Only 15% reported a decrease. A qualitative analysis of responses from over 500 Australian healthcare workers during the pandemic identified a central theme of staff being bullied, intimidated, and censured by colleagues and hospital management [43]. Such accounts would suggest that, a priori, an increase in unprofessional behaviours at follow-up may have been expected because of increased work demands, which is the opposite to our finding.

We found that non-clinical and management respondents experienced the greatest reduction in incivility/bullying. Some of these staff may have had greater opportunity to work remotely during the pandemic and thus have had reduced interaction with other staff. This group included a broad range of personnel, including laboratory scientists, cleaners and catering staff, many of whom were unable to work from home. Certainly, the importance of roles such as cleaners in infection control became more prominent during the pandemic [44] and this may have also improved general staff behaviours towards these individuals.

Other than the pandemic, we have no information to suggest that there was a significant temporal improvement in unprofessional behaviours across the health system. To the contrary, a 2021 report of medical trainees showed bullying and harassment continued to occur at high rates [45]. However, we cannot rule out other possible factors contributed to the reported decrease in unprofessional behaviours experienced.

As expected, we received a lower response rate at follow-up given it was conducted in 2021/2022 when the effects of the pandemic continued. In particular, we had a low response rate from medical staff. We compared the characteristics of the baseline and follow-up samples, adjusted for these factors, and undertook subgroup analyses with and without medical respondents for the key outcomes to assess the impact on the results. In our analysis of Ethos submissions across eight hospitals [28], we found similar rates of use of the Ethos reporting system (14.1 submissions/100 staff) as reported by those who responded to the survey, which indicates survey respondents had similar levels of engagement with the Program. The most frequent unprofessional behaviours reported to the messaging system were consistent with the most frequent behaviours reported in the staff surveys. Thus, behaviours experienced by staff which motivate them to complete an Ethos reflection submission, are largely the same unprofessional behaviours staff report when asked by survey. This result lends support to the validity of staff surveys in reflecting the nature and prevalence of unprofessional behaviours experienced. We have no information that indicates staff motivations for completing the baseline and follow-up surveys were different.

Overall, the Ethos program evaluation results provide several types of qualitative and quantitative evidence that staff engaged with the program, that it permeated throughout the different professional groups through different mechanisms and influenced behaviours. Significant culture change is very difficult. This final element of the evaluation provides some promising findings that the program may be having some positive effects and continued support for the program is warranted. Future studies could also consider staff turnover, psychosocial workplace injury claims, and sick leave rates among staff as further indicators of program effectiveness.

## Conclusions

Implementation of the Ethos program was associated with significant reductions in the rate at which hospital staff reported experiencing unprofessional behaviours. Findings from our evaluation program add to the evidence-base indicating that professional accountability programs may be effective in improving organisational cultures by reducing unprofessional behaviours. Importantly our results suggest two areas for policy makers to enhance interventions, namely, by encouraging both feedback for reflection and recognition; and by ensuring interventions are available to all hospital staff, and not focused on siloed professional groups.

Evaluation of long-term organisational culture change programs are methodologically difficult and are rare in the literature. However, these challenges should not dissuade researchers and organisations from pursuing evidence to inform the continued development of effective organisational change strategies.

### Supplementary Information

Supplementary Material 1.

Supplementary Material 2.

Supplementary Material 3.

Supplementary Material 4.

Supplementary Material 5.

Supplementary Material 6.

Supplementary Material 7.

## Data Availability

No datasets were generated or analysed during the current study.
