# Supplementary material for: Evaluation of a culture change program to reduce unprofessional behaviours by hospital co-workers in Australian hospitals
Source: BMC Health Serv Res. 2024 Jun 12;24:722. doi: 10.1186/s12913-024-11171-0 (PMC11167838; doi:10.1186/s12913-024-11171-0)
Supplement: Supplementary file 1 — Supplementary Material 1. [file 12913_2024_11171_MOESM1_ESM.docx]

**Supplementary File 1. Longitudinal Investigation Of Negative behaviour (LION) Survey questions relevant to this report and response rates by role.**

**Demographics**

**Age:** ❑ 18-24 ❑ 25-34 ❑ 35-44 ❑ 45-54 ❑ 55-64 ❑ 65+

**Gender:** ❑ Female ❑ Male ❑ Other ❑ Prefer not to answer

**Length of employment at current hospital:** ❑ Less than 1 year ❑ 1-2 years ❑ 3-5 years ❑ 6-10 years ❑ 11-20 years ❑ Over 20 years

**Length of employment in the healthcare sector:** ❑ Less than 1 year ❑ 1-2 years ❑ 3-5 years ❑ 6-10 years ❑ 11-20 years ❑ Over 20 years

**Hospital:** ❑ A ❑ B ❑ C ❑ D ❑ E

**What is your main role at St Vincent’s?**

| **Medical**  ❑ Surgical Staff specialist/ VMO  ❑ Medical Staff specialist/ VMO  ❑ Registrar  ❑ Career/Hospital Medical Officer/Medical Fellow  ❑ Resident  ❑ Intern | **Nursing**  ❑ Nurse Unit Manager or Associate NUM  ❑ Clinical Nurse Consultant/Specialist/ Educator  ❑ Registered nurse or midwife  ❑ Enrolled nurse  ❑ Graduate nurse or midwife | **Allied Health & Clinical Services**  ❑ Allied health (e.g. pharmacy, physiotherapy, occupational therapy, dietitian)  ❑ Clinical services (e.g. psychology, medical imaging, perfusionist, technologist, pathology collector)  ❑ Social, welfare or pastoral care worker  ❑ Other clinical services | **Non-clinical Services**  ❑ Scientist, laboratory or research staff  ❑ Personal care/patient services assistant or orderly  ❑ Food services  ❑ Engineering services, security or tradesperson  ❑ Cleaner/environmental services  ❑ Other non-clinical staff | **Management & Administrative**  ❑ Administrative staff (Human Resources, medical records, IT, finance officer)  ❑ Manager  ❑ Ward Clerk, patient services Clerk  ❑ Other management & administrative |
| --- | --- | --- | --- | --- |

**Twenty-six unprofessional behaviours**

**We are interested in behaviours exhibited by other staff towards you. We are NOT asking about times when you have seen patients or other hospital visitors exhibit these behaviours.**

| In the past 12 months, how often have you experienced the following staff behaviours in this hospital? | Frequency | | | | | | | | | | What professional group did the person(s) who engaged in this behaviour belong to? | | | | | | Please indicate their professional seniority level. | | | | |  |  |
| --- | --- | --- | --- | --- | --- | --- | --- | --- | --- | --- | --- | --- | --- | --- | --- | --- | --- | --- | --- | --- | --- | --- | --- |
|  | **Never** | **1-2 times/ year** | **Every few months** | | **Around**  **monthly** | **Weekly** | | **Daily** | | **Multiple times daily** | **Medical** | **Nursing** | **Allied Health & Clinical Services** | **Non-Clinical Services** | **Management and Administrative** | **Prefer not to**  **say** | **Senior to me** | **Same level as me** | | | **Junior to me** |  |  |
| Being spoken to rudely | ❑ | ❑ | ❑ | | ❑ | ❑ | | ❑ | ❑ | | ❑ | ❑ | ❑ | ❑ | ❑ | ❑ | ❑ | | ❑ | ❑ | |  |  |
| Someone withholding information which affects work performance | ❑ | ❑ | ❑ | | ❑ | ❑ | | ❑ | ❑ | | ❑ | ❑ | ❑ | ❑ | ❑ | ❑ | ❑ | | ❑ | ❑ | |  |  |
| Opinions being ignored | ❑ | ❑ | ❑ | | ❑ | ❑ | | ❑ | ❑ | | ❑ | ❑ | ❑ | ❑ | ❑ | ❑ | ❑ | | ❑ | ❑ | |  |  |
| Being shouted at or being the target of anger | ❑ | ❑ | ❑ | | ❑ | ❑ | | ❑ | ❑ | | ❑ | ❑ | ❑ | ❑ | ❑ | ❑ | ❑ | | ❑ | ❑ | |  |  |
| Being told sexually explicit or offensive jokes/ comments at work | ❑ | ❑ | ❑ | | ❑ | ❑ | | ❑ | ❑ | | ❑ | ❑ | ❑ | ❑ | ❑ | ❑ | ❑ | | ❑ | ❑ | |  |  |
| Physically intimidating behaviours (e.g. finger-pointing, invasion of personal space, blocking) | ❑ | ❑ | ❑ | | ❑ | ❑ | | ❑ | ❑ | | ❑ | ❑ | ❑ | ❑ | ❑ | ❑ | ❑ | | ❑ | ❑ | |  |  |
| Hints or signals from others to quit your job | ❑ | ❑ | ❑ | | ❑ | ❑ | | ❑ | ❑ | | ❑ | ❑ | ❑ | ❑ | ❑ | ❑ | ❑ | | ❑ | ❑ | |  |  |
| Repeated reminders of errors or mistakes | ❑ | ❑ | ❑ | | ❑ | ❑ | | ❑ | ❑ | | ❑ | ❑ | ❑ | ❑ | ❑ | ❑ | ❑ | | ❑ | ❑ | |  |  |
| Excessive monitoring of work | ❑ | ❑ | ❑ | | ❑ | ❑ | | ❑ | ❑ | | ❑ | ❑ | ❑ | ❑ | ❑ | ❑ | ❑ | | ❑ | ❑ | |  |  |
| Unwelcome practical jokes | ❑ | ❑ | ❑ | | ❑ | ❑ | | ❑ | ❑ | | ❑ | ❑ | ❑ | ❑ | ❑ | ❑ | ❑ | | ❑ | ❑ | |  |  |
| Being given unreasonable workload/deadlines | ❑ | ❑ | ❑ | | ❑ | ❑ | | ❑ | ❑ | | ❑ | ❑ | ❑ | ❑ | ❑ | ❑ | ❑ | | ❑ | ❑ | |  |  |
| Graphic comments/ questions/ insinuations about appearance, sexual or private life | ❑ | ❑ | ❑ | | ❑ | ❑ | | ❑ | ❑ | | ❑ | ❑ | ❑ | ❑ | ❑ | ❑ | ❑ | | ❑ | ❑ | |  |  |
| Being the subject of excessive teasing/sarcasm | ❑ | ❑ | ❑ | | ❑ | ❑ | | ❑ | ❑ | | ❑ | ❑ | ❑ | ❑ | ❑ | ❑ | ❑ | | ❑ | ❑ | |  |  |
| Threats of violence/physical abuse | ❑ | ❑ | ❑ | | ❑ | ❑ | | ❑ | ❑ | | ❑ | ❑ | ❑ | ❑ | ❑ | ❑ | ❑ | | ❑ | ❑ | |  |  |
| Being ignored or excluded | ❑ | ❑ | ❑ | | ❑ | ❑ | | ❑ | ❑ | | ❑ | ❑ | ❑ | ❑ | ❑ | ❑ | ❑ | | ❑ | ❑ | |  |  |
| Inappropriate or unwanted touching | ❑ | ❑ | ❑ | | ❑ | ❑ | | ❑ | ❑ | | ❑ | ❑ | ❑ | ❑ | ❑ | ❑ | ❑ | | ❑ | ❑ | |  |  |
| Unwelcome sexual flirtations/persistent requests for dates | ❑ | ❑ | ❑ | | ❑ | ❑ | | ❑ | ❑ | | ❑ | ❑ | ❑ | ❑ | ❑ | ❑ | ❑ | | ❑ | ❑ | |  |  |
| Being humiliated or ridiculed | ❑ | ❑ | ❑ | | ❑ | ❑ | | ❑ | ❑ | | ❑ | ❑ | ❑ | ❑ | ❑ | ❑ | ❑ | | ❑ | ❑ | |  |  |
| Demands for sexual favours | ❑ | ❑ | ❑ | | ❑ | ❑ | | ❑ | ❑ | | ❑ | ❑ | ❑ | ❑ | ❑ | ❑ | ❑ | | ❑ | ❑ | |  |  |
| Having unjustified allegations made | ❑ | ❑ | ❑ | | ❑ | ❑ | | ❑ | ❑ | | ❑ | ❑ | ❑ | ❑ | ❑ | ❑ | ❑ | | ❑ | ❑ | |  |  |
| Having key areas of responsibility removed or replaced with meaningless or unpleasant tasks | ❑ | ❑ | ❑ | | ❑ | ❑ | | ❑ | ❑ | | ❑ | ❑ | ❑ | ❑ | ❑ | ❑ | ❑ | | ❑ | ❑ | |  |  |
| Being shown sexually suggestive photos, videos, emails or texts | ❑ | ❑ | ❑ | | ❑ | ❑ | | ❑ | ❑ | | ❑ | ❑ | ❑ | ❑ | ❑ | ❑ | ❑ | | ❑ | ❑ | |  |  |
| Sexual assault | ❑ | ❑ | ❑ | | ❑ | ❑ | | ❑ | ❑ | | ❑ | ❑ | ❑ | ❑ | ❑ | ❑ | ❑ | | ❑ | ❑ | |  |  |
| Physical assault (e.g. hitting, shoving, punching) | ❑ | ❑ | ❑ | | ❑ | ❑ | | ❑ | ❑ | | ❑ | ❑ | ❑ | ❑ | ❑ | ❑ | ❑ | | ❑ | ❑ | |  |  |
| Negative comments or offensive jokes about gender, ethnicity, sexual orientation, religion, disability, pregnancy, parenting responsibilities | ❑ | ❑ | ❑ | | ❑ | ❑ | | ❑ | ❑ | | ❑ | ❑ | ❑ | ❑ | ❑ | ❑ | ❑ | | ❑ | ❑ | |  |  |
| *If yes, comments/jokes were based on (choose all that apply):* | Gender ⭘ | | | Ethnicity ⭘ | | | Sexual orientation ⭘ | Religion ⭘ | | |  | | | | | |  | | | | |  |  |
|  | Disability ⭘ | | | Pregnancy ⭘ | | | Parenting/carer responsibilities ⭘ | | | |  |  |  |  |  |  |  | | | | |  |  |
| Treated unfairly based on gender, ethnicity, sexual orientation, religion, disability, pregnancy, parenting responsibilities | ❑ | ❑ | ❑ | | ❑ | ❑ | | ❑ | ❑ | | ❑ | ❑ | ❑ | ❑ | ❑ | ❑ | ❑ | | ❑ | ❑ | |  |  |
| *If yes, unfair treatment was based on (choose all that apply):* | Gender ⭘ | | | Ethnicity ⭘ | | | Sexual orientation ⭘ | Religion ⭘ | | |  | | | | | |  | | | | |  |  |
|  | Disability ⭘ | | | Pregnancy ⭘ | | | Parenting/carer responsibilities ⭘ | | | |  |  |  |  |  |  |  | | | | |  |  |

**Speaking up**

| Thinking about unprofessional staff behaviours in this hospital, how much do you agree or disagree with the following statements: | Strongly disagree | Disagree | Neither disagree nor agree | Agree | Strongly agree | Prefer not to answer |
| --- | --- | --- | --- | --- | --- | --- |
| Speaking up or reporting unprofessional behaviour is important for patient safety | ❑ | ❑ | ❑ | ❑ | ❑ | ❑ |
| I am encouraged by my colleagues to speak up about unprofessional behaviour | ❑ | ❑ | ❑ | ❑ | ❑ | ❑ |
| I have the skills to effectively speak up if I experience unprofessional behaviour | ❑ | ❑ | ❑ | ❑ | ❑ | ❑ |
| I have the skills to effectively speak up if others experience unprofessional behaviour | ❑ | ❑ | ❑ | ❑ | ❑ | ❑ |
| I know the proper channels to raise concerns about unprofessional behaviour | ❑ | ❑ | ❑ | ❑ | ❑ | ❑ |
| Unprofessional behaviour is effectively managed in this hospital | ❑ | ❑ | ❑ | ❑ | ❑ | ❑ |
| I feel comfortable speaking up or reporting unprofessional behaviour | ❑ | ❑ | ❑ | ❑ | ❑ | ❑ |
| It takes too much time and effort to report unprofessional behaviour | ❑ | ❑ | ❑ | ❑ | ❑ | ❑ |
| I am confident I would receive support from my supervisor if I reported unprofessional behaviour | ❑ | ❑ | ❑ | ❑ | ❑ | ❑ |
| Speaking up or reporting unprofessional behaviour is likely to have a negative impact on my career | ❑ | ❑ | ❑ | ❑ | ❑ | ❑ |
| I am confident I would be believed and taken seriously if I reported unprofessional behaviour | ❑ | ❑ | ❑ | ❑ | ❑ | ❑ |

**Covid-19**

|  | Decreased | Increased | Did not change |
| --- | --- | --- | --- |
| During the Covid-19 pandemic, unprofessional behaviour… | ❑ | ❑ | ❑ |
|  | **Small** | **Moderate** | **Large** |
| The increase in unprofessional behaviour during the Covid-19 pandemic was... | ❑ | ❑ | ❑ |
|  | **Small** | **Moderate** | **Large** |
| The increase in unprofessional behaviour during the Covid-19 pandemic was... | ❑ | ❑ | ❑ |
|  | **Yes** | **No** | **Unsure** |
| During the Covid-19 pandemic, did teamwork and cooperation improve? | ❑ | ❑ | ❑ |
|  | **Was a small improvement** | **Was a moderate improvement** | **Was a large improvement** |
| The improvement in teamwork and cooperation during the Covid-19  pandemic... |  |  |  |
| Within my professional group | ❑ | ❑ | ❑ |
| Between professional groups | ❑ | ❑ | ❑ |
|  | **Yes** | **No** | **Unsure** |
| During the Covid-19 pandemic, did teamwork and cooperation decrease? | ❑ | ❑ | ❑ |
|  | **Was a small decrease** | **Was a moderate decrease** | **Was a large decrease** |
| The decrease in teamwork and cooperation during the Covid-19  pandemic... |  |  |  |
| Within my professional group | ❑ | ❑ | ❑ |
| Between professional groups | ❑ | ❑ | ❑ |

**Ethos**

**Are you aware of the *Ethos* Program at SVHA**: ❑ Yes ❑ No

|  | Yes | No |
| --- | --- | --- |
| I have… |  |  |
| Seen Ethos promotional material | ❑ | ❑ |
| Completed the Ethos training | ❑ | ❑ |
| Used the Ethos messaging system to provide recognition or feedback | ❑ | ❑ |
| Received an Ethos message | ❑ | ❑ |
| Been an Ethos messenger | ❑ | ❑ |
| Changed the way I interact with other staff as a result of the Ethos program | ❑ | ❑ |

|  | Positive (Recognition) | Negative (Reflection) |
| --- | --- | --- |
| The type of *Ethos*message I received was... | ❑ | ❑ |

|  | Strongly disagree | Disagree | Neither agree nor disagree | Agree | Strongly agree |
| --- | --- | --- | --- | --- | --- |
| The Ethos program demonstrates a commitment to improving workplace culture in this hospital | ❑ | ❑ | ❑ | ❑ | ❑ |

|  | No impact | Minor impact | Moderate impact | Major impact | Not sure |  |
| --- | --- | --- | --- | --- | --- | --- |
| To what extent has the Ethos program positively impacted… |  |  |  |  |  |  |
| You and your wellbeing? | ❑ | ❑ | ❑ | ❑ | ❑ |  |
| Other staff and their wellbeing? | ❑ | ❑ | ❑ | ❑ | ❑ |  |
| Your ability to speak up about yourself? | ❑ | ❑ | ❑ | ❑ | ❑ |  |
| Your ability to speak up for others? | ❑ | ❑ | ❑ | ❑ | ❑ |  |
| The ability of other staff to speak up? | ❑ | ❑ | ❑ | ❑ | ❑ |  |
| Patient care? | ❑ | ❑ | ❑ | ❑ | ❑ |  |
| Teamwork? | | ❑ | ❑ | ❑ | ❑ | ❑ |
| Frequency of errors or mistakes? | | ❑ | ❑ | ❑ | ❑ | ❑ |
| Quality of service provided at this hospital? | | ❑ | ❑ | ❑ | ❑ | ❑ |

|  | Not at all effective | Not very effective | Slightly effective | Moderately effective | Completely effective | Not sure |
| --- | --- | --- | --- | --- | --- | --- |
| How effective do you believe the Ethos program has been at reducing unprofessional behaviours… |  |  |  |  |  |  |
| 1. In your profession? | ❑ | ❑ | ❑ | ❑ | ❑ | ❑ |
| 1. In this hospital? | ❑ | ❑ | ❑ | ❑ | ❑ | ❑ |

**Response rates by role at baseline and followup**

|  | Baseline |  | Follow-up |  |
| --- | --- | --- | --- | --- |
| Role | n/N | response rate (%) | n/N | response rate (%) |
| Nursing | 1035/3926 | 26.4 | 637/4089 | 15.6 |
| Medical | 302/1008 | 30.0 | 75/905 | 8.3 |
| Allied Health & Clinical Services | 371/841 | 44.1 | 214/987 | 21.7 |
| Non-clinical Services | 337/1382 | 24.4 | 211/1590 | 13.3 |
| Management & Administrative | 423/1163 | 36.4 | 284/802 | 35.4 |
| Missing | (84) |  | (2) |  |
| Overall | 2552/8320 | 30.7 | 1423/8373 | 17.0 |

**Survey completion data**

| **Time points** | **Total number of respondents** | **Number of respondents included in the study (%)** | **Average % of completion across included respondents (SD)** | **Median % of completion across included respondents (IQR)** |
| --- | --- | --- | --- | --- |
| **Baseline** | 2790 | 2552 (91.5%) | 98.1% (5.4) | 100% (98.8-100) |
| **Follow-up** | 1583 | 1423 (89.9%) | 95.8% (8.6) | 100% (97.7-100) |
