# Supplementary material for: Evaluation of a culture change program to reduce unprofessional behaviours by hospital co-workers in Australian hospitals
Source: BMC Health Serv Res. 2024 Jun 12;24:722. doi: 10.1186/s12913-024-11171-0 (PMC11167838; doi:10.1186/s12913-024-11171-0)
Supplement: Supplementary file 2 — Supplementary Material 2. [file 12913_2024_11171_MOESM2_ESM.docx]

Supplementary File 2. Odds ratios for experiencing incivility/bullying at each hospital, follow-up vs baseline, excluding Medical respondents.

| Hospital | Number of responses  (baseline and follow-up) | Unadjusted analysis | | Adjusted analysis* | |
| --- | --- | --- | --- | --- | --- |
|  |  | OR | 95% CI | aOR | 95% CI |
| Hospital A | 1462 | 0.77 | 0.62, 0.96 | 0.80 | 0.64, 1.00 |
| Hospital B | 400 | 0.73 | 0.49, 1.09 | 0.72 | 0.48, 1.10 |
| Hospital C | 528 | 0.51 | 0.36, 0.73 | 0.54 | 0.38, 0.77 |
| Hospital D | 635 | 0.74 | 0.54, 1.00 | 0.74 | 0.53, 1.02 |
| Hospital E | 223 | 0.88 | 0.54, 1.45 | 0.86 | 0.51, 1.45 |

* Adjusted for age, gender, hospital, and length of employment in the hospital and in the sector. ORs - Odds ratios are from a proportional odds model comparing odds of experiencing unprofessional behaviour Occasionally vs Never or odds of experiencing unprofessional behaviour Frequently vs Occasionally/Never at follow-up compared with baseline.
