# Supplementary material for: Evaluation of a culture change program to reduce unprofessional behaviours by hospital co-workers in Australian hospitals
Source: BMC Health Serv Res. 2024 Jun 12;24:722. doi: 10.1186/s12913-024-11171-0 (PMC11167838; doi:10.1186/s12913-024-11171-0)
Supplement: Supplementary file 3 — Supplementary Material 3. [file 12913_2024_11171_MOESM3_ESM.docx]

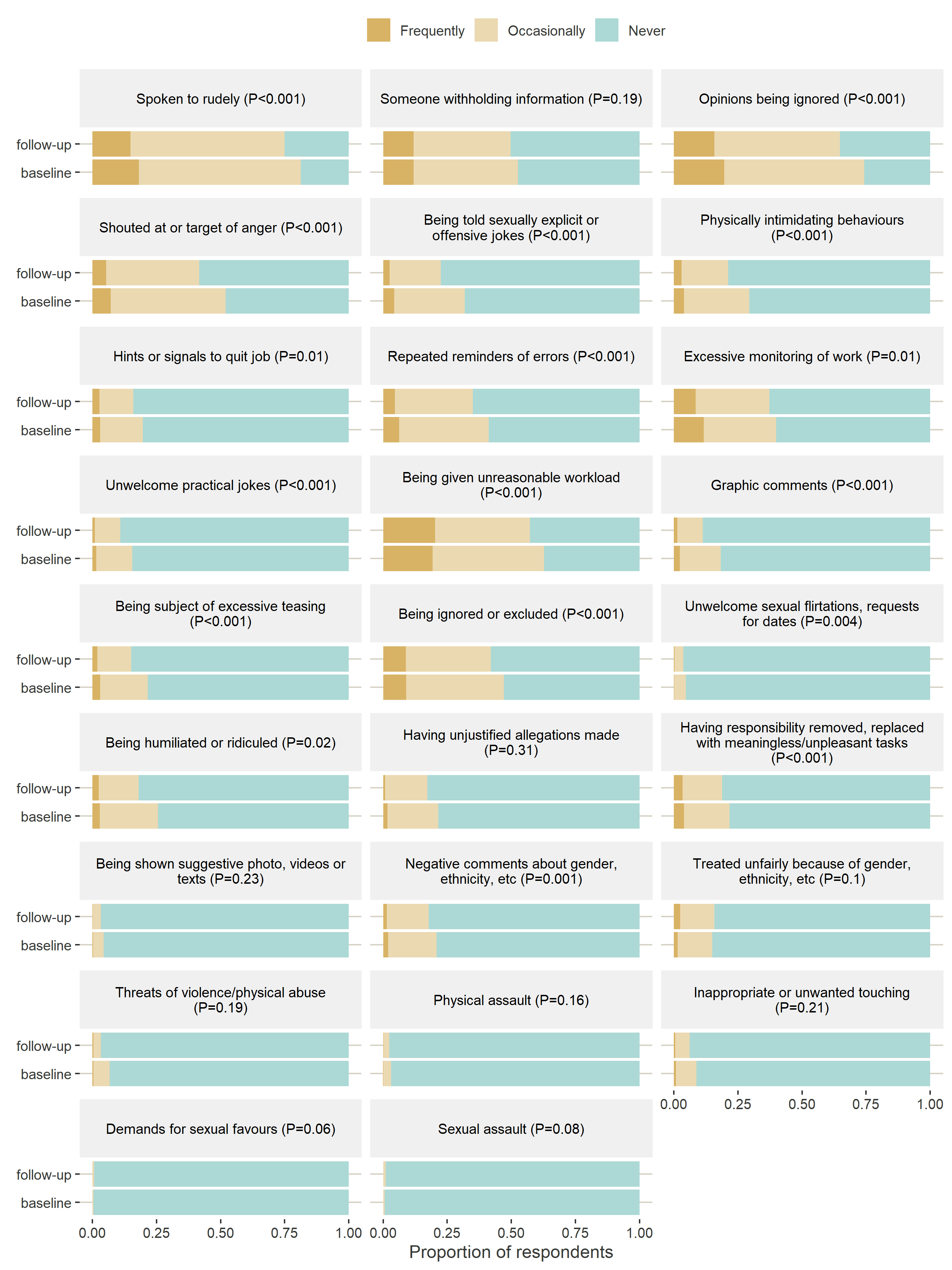


**Supplementary File 3. Comparison of the proportions of staff reporting each of the 26 unprofessional behaviours at baseline and follow-up.**
