# Supplementary material for: Evaluation of a culture change program to reduce unprofessional behaviours by hospital co-workers in Australian hospitals
Source: BMC Health Serv Res. 2024 Jun 12;24:722. doi: 10.1186/s12913-024-11171-0 (PMC11167838; doi:10.1186/s12913-024-11171-0)
Supplement: Supplementary file 4 — Supplementary Material 4. [file 12913_2024_11171_MOESM4_ESM.docx]

Supplementary File 4. Odds ratios for experiencing extreme unprofessional behaviour at each hospital, follow-up vs baseline.

**A: Including Medical respondents**

| Hospital | Number of responses  (baseline and follow-up) | Unadjusted analysis | | Adjusted analysis* | |
| --- | --- | --- | --- | --- | --- |
|  |  | OR | 95% CI | OR | 95% CI |
| Hospital A | 1712 | 0.65 | 0.47, 0.90 | 0.66 | 0.47, 0.91 |
| Hospital B | 428 | 0.92 | 0.50, 1.67 | 1.11 | 0.58, 2.14 |
| Hospital C | 608 | 1.25 | 0.68, 2.31 | 1.24 | 0.63, 2.44 |
| Hospital D | 690 | 0.43 | 0.26, 0.71 | 0.42 | 0.25, 0.72 |
| Hospital E | 247 | 0.68 | 0.30, 1.52 | 0.67 | 0.26, 1.75 |

* Adjusted for age, gender, role, and length of employment in the hospital and in the sector.

**B: Excluding Medical respondents**

| Hospital | Number of responses  (baseline and follow-up) | Unadjusted analysis | | Adjusted analysis* | |
| --- | --- | --- | --- | --- | --- |
|  |  | OR | 95% CI | OR | 95% CI |
| Hospital A | 1501 | 0.65 | 0.46, 0.90 | 0.65 | 0.46, 0.91 |
| Hospital B | 410 | 0.93 | 0.51, 1.70 | 1.11 | 0.58, 2.14 |
| Hospital C | 536 | 1.09 | 0.58, 2.05 | 1.27 | 0.65, 2.48 |
| Hospital D | 654 | 0.38 | 0.22, 0.64 | 0.38 | 0.22, 0.66 |
| Hospital E | 227 | 0.82 | 0.35, 1.92 | 0.82 | 0.30, 2.20 |

* Adjusted for age, gender, role, and length of employment in the hospital and in the sector.
