# Supplementary material for: Evaluation of a culture change program to reduce unprofessional behaviours by hospital co-workers in Australian hospitals
Source: BMC Health Serv Res. 2024 Jun 12;24:722. doi: 10.1186/s12913-024-11171-0 (PMC11167838; doi:10.1186/s12913-024-11171-0)

**Supplementary File 5. Relative seniority of perpetrators of unprofessional behaviour compared with victim.**
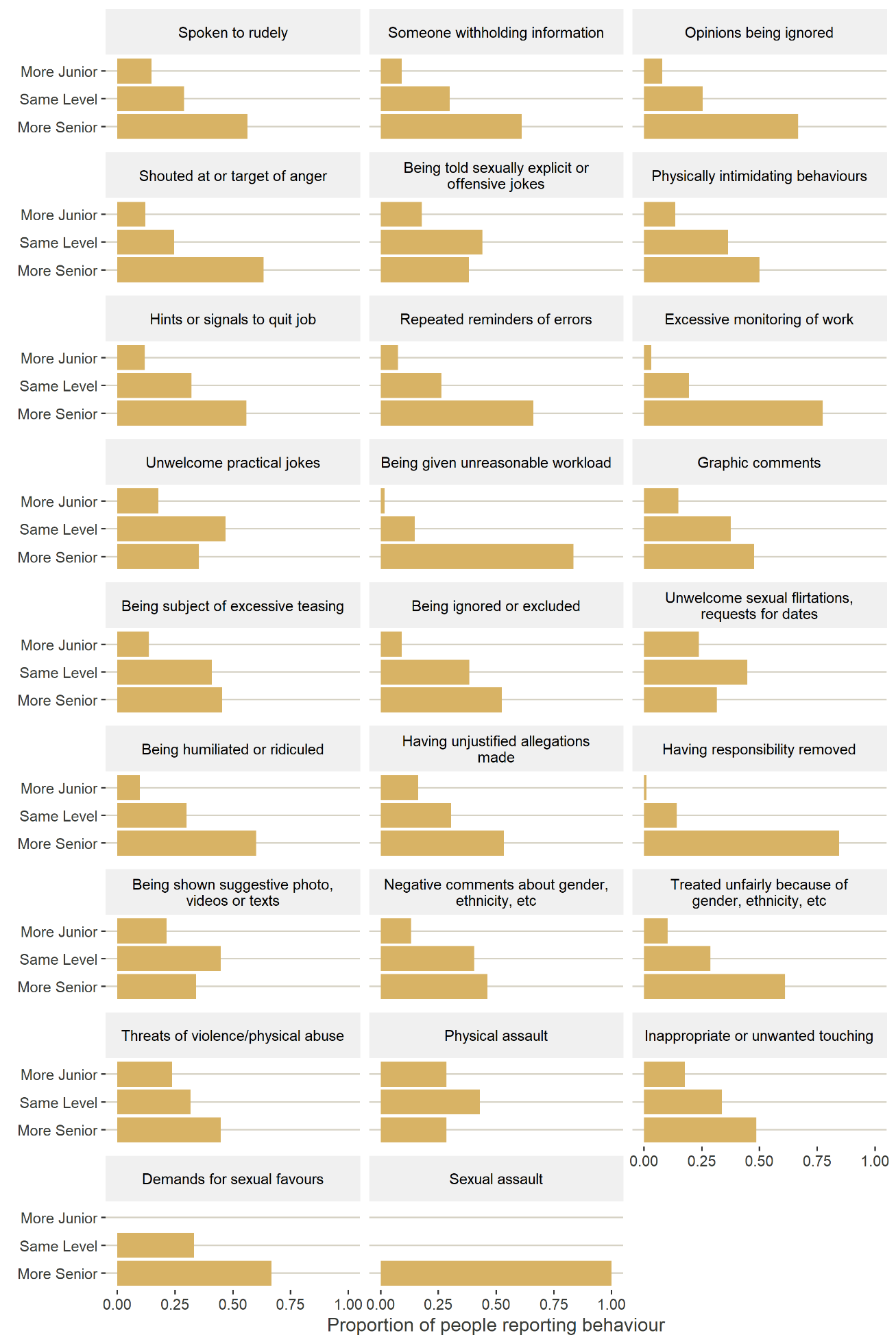

Supplement: Supplementary file 5 — Supplementary Material 5. [file 12913_2024_11171_MOESM5_ESM.docx]
