# Supplementary material for: Evaluation of a culture change program to reduce unprofessional behaviours by hospital co-workers in Australian hospitals
Source: BMC Health Serv Res. 2024 Jun 12;24:722. doi: 10.1186/s12913-024-11171-0 (PMC11167838; doi:10.1186/s12913-024-11171-0)
Supplement: Supplementary file 6 — Supplementary Material 6. [file 12913_2024_11171_MOESM6_ESM.docx]

**Supplementary File 6. Speaking-up and reporting unprofessional behaviours.**

|  | Strongly Disagree | | Disagree | | Neither Disagree nor Agree | | Agree | | Strongly Agree | | Negative | | Neutral | | Positive | | Missing |
| --- | --- | --- | --- | --- | --- | --- | --- | --- | --- | --- | --- | --- | --- | --- | --- | --- | --- |
|  | N | % | N | % | N | % | N | % | N | % | N | % | N | % | N | % | N |
| ***Speaking up or reporting unprofessional behaviour is important for patient safety (p<0.001)*** | | | | | | | | | | |  |  |  |  |  |  |  |
| baseline | 88 | 3.6 | 11 | 0.4 | 108 | 4.4 | 769 | 31.2 | 1488 | 60.4 | 99 | 4.0 | 108 | 4.4 | 2257 | 91.6 | 88 |
| follow-up | 37 | 2.8 | 11 | 0.8 | 53 | 4.0 | 327 | 24.6 | 901 | 67.8 | 48 | 3.6 | 53 | 4.0 | 1228 | 92.4 | 94 |
| ***I am encouraged by my colleagues to speak up about unprofessional behaviour (p<0.001)*** | | | | | | | | | | |  |  |  |  |  |  |  |
| baseline | 149 | 6.1 | 288 | 11.7 | 470 | 19.1 | 826 | 33.6 | 726 | 29.5 | 437 | 17.8 | 470 | 19.1 | 1552 | 63.1 | 93 |
| follow-up | 58 | 4.4 | 125 | 9.4 | 210 | 15.8 | 466 | 35.1 | 467 | 35.2 | 183 | 13.8 | 210 | 15.8 | 933 | 70.4 | 97 |
| ***I have the skills to effectively speak up if I experience unprofessional behaviour (p=0.01)*** | | | | | | | | | | |  |  |  |  |  |  |  |
| baseline | 84 | 3.4 | 218 | 8.9 | 283 | 11.5 | 1056 | 43.0 | 814 | 33.2 | 302 | 12.3 | 283 | 11.5 | 1870 | 76.2 | 97 |
| follow-up | 25 | 1.9 | 91 | 6.9 | 158 | 11.9 | 581 | 43.8 | 473 | 35.6 | 116 | 8.7 | 158 | 11.9 | 1054 | 79.4 | 95 |
| ***I have the skills to effectively speak up if others experience unprofessional behaviour (p<0.001)*** | | | | | | | | | | |  |  |  |  |  |  |  |
| baseline | 79 | 3.2 | 211 | 8.6 | 344 | 14.0 | 1093 | 44.6 | 725 | 29.6 | 290 | 11.8 | 344 | 14.0 | 1818 | 74.1 | 100 |
| follow-up | 25 | 1.9 | 68 | 5.1 | 148 | 11.2 | 601 | 45.3 | 484 | 36.5 | 93 | 7.0 | 148 | 11.2 | 1085 | 81.8 | 97 |
| ***I know the proper channels to raise concerns about unprofessional behaviour (p<0.001)*** | | | | | | | | | | |  |  |  |  |  |  |  |
| baseline | 116 | 4.7 | 218 | 8.8 | 273 | 11.1 | 1090 | 44.2 | 768 | 31.2 | 334 | 13.5 | 273 | 11.1 | 1858 | 75.4 | 87 |
| follow-up | 38 | 2.9 | 75 | 5.6 | 135 | 10.2 | 577 | 43.4 | 505 | 38.0 | 113 | 8.5 | 135 | 10.2 | 1082 | 81.4 | 93 |
| ***Unprofessional behaviour is effectively managed in this hospital (p=0.2)*** | | | | | | | | | | |  |  |  |  |  |  |  |
| baseline | 357 | 14.7 | 517 | 21.3 | 635 | 26.2 | 606 | 25.0 | 313 | 12.9 | 874 | 36.0 | 635 | 26.2 | 919 | 37.9 | 124 |
| follow-up | 172 | 13.1 | 253 | 19.3 | 381 | 29.0 | 334 | 25.5 | 172 | 13.1 | 425 | 32.4 | 381 | 29.0 | 506 | 38.6 | 111 |
| ***I feel comfortable speaking up or reporting unprofessional behaviour (p=0.017)*** | | | | | | | | | | |  |  |  |  |  |  |  |
| baseline | 186 | 7.6 | 483 | 19.8 | 395 | 16.2 | 911 | 37.3 | 467 | 19.1 | 669 | 27.4 | 395 | 16.2 | 1378 | 56.4 | 110 |
| follow-up | 117 | 8.8 | 220 | 16.6 | 229 | 17.3 | 464 | 35.0 | 294 | 22.2 | 337 | 25.5 | 229 | 17.3 | 758 | 57.3 | 99 |
| ***It takes too much time and effort to report unprofessional behaviour (p=0.1)*** | | | | | | | | | | |  |  |  |  |  |  |  |
| baseline | 257 | 10.5 | 764 | 31.3 | 668 | 27.4 | 553 | 22.7 | 197 | 8.1 | 1021 | 41.9 | 668 | 27.4 | 750 | 30.8 | 113 |
| follow-up | 134 | 10.2 | 364 | 27.6 | 382 | 29.0 | 313 | 23.7 | 125 | 9.5 | 498 | 37.8 | 382 | 29.0 | 438 | 33.2 | 105 |
| ***I am confident I would receive support from my supervisor if I reported unprofessional behaviour (p=0.019)*** | | | | | | | | | | | | | | | | | |
| baseline | 225 | 9.2 | 265 | 10.9 | 505 | 20.7 | 881 | 36.1 | 563 | 23.1 | 490 | 20.1 | 505 | 20.7 | 1444 | 59.2 | 113 |
| follow-up | 114 | 8.6 | 168 | 12.7 | 234 | 17.8 | 452 | 34.3 | 350 | 26.6 | 282 | 21.4 | 234 | 17.8 | 802 | 60.8 | 105 |
| ***Speaking up or reporting unprofessional behaviour is likely to have a negative impact on my career (p=0.8)*** | | | | | | | | | | |  |  |  |  |  |  |  |
| baseline | 217 | 8.9 | 653 | 26.8 | 709 | 29.0 | 554 | 22.7 | 308 | 12.6 | 870 | 35.6 | 709 | 29.0 | 862 | 35.3 | 111 |
| follow-up | 116 | 8.8 | 338 | 25.8 | 369 | 28.1 | 307 | 23.4 | 182 | 13.9 | 454 | 34.6 | 369 | 28.1 | 489 | 37.3 | 111 |
| ***I am confident I would be believed and taken seriously if I reported unprofessional behaviour (p=0.1)*** | | | | | | | | | | |  |  |  |  |  |  |  |
| baseline | 150 | 6.2 | 285 | 11.7 | 551 | 22.6 | 995 | 40.9 | 453 | 18.6 | 435 | 17.9 | 551 | 22.6 | 1448 | 59.5 | 118 |
| follow-up | 80 | 6.0 | 135 | 10.2 | 331 | 25.0 | 503 | 38.0 | 274 | 20.7 | 215 | 16.3 | 331 | 25.0 | 777 | 58.7 | 100 |

“Negative” = Strongly Disagree + Disagree. “Neutral” = Neither Disagree nor Agree. “Positive” = Agree + Strongly Agree.
