# Supplementary figures and images for: Evaluation of a culture change program to reduce unprofessional behaviours by hospital co-workers in Australian hospitals
Source: BMC Health Serv Res. 2024 Jun 12;24:722. doi: 10.1186/s12913-024-11171-0 (PMC11167838; doi:10.1186/s12913-024-11171-0)

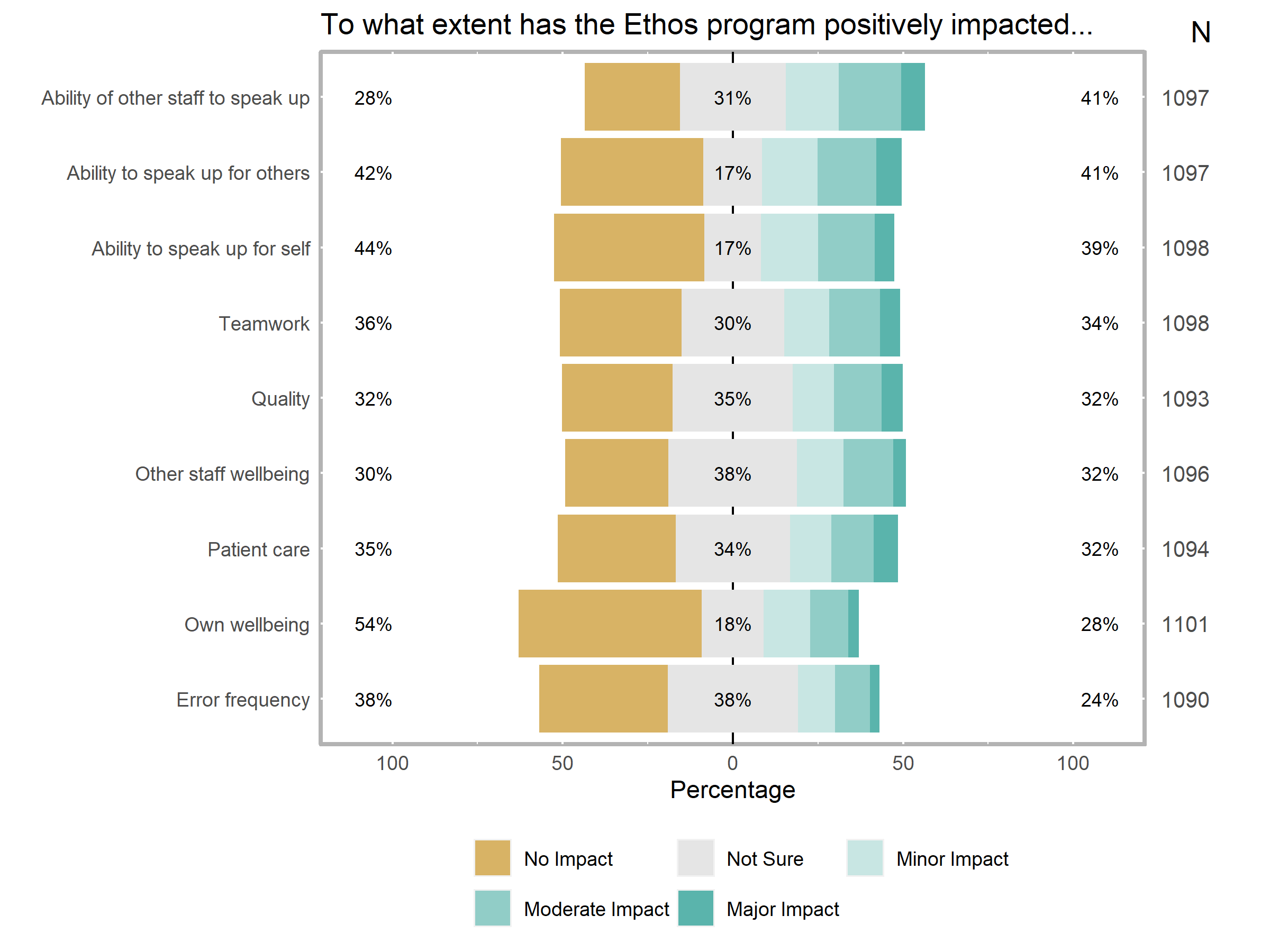


**Supplementary File 7. Perceived impact of Ethos**

Supplement: Supplementary file 7 — Supplementary Material 7. [file 12913_2024_11171_MOESM7_ESM.docx]
